# Precision ophthalmology: a call for Africa not to be left in the dark

**DOI:** 10.1038/s41434-024-00448-y

**Published:** 2024-03-22

**Authors:** Lisa Roberts

**Affiliations:** UCT/MRC Precision and Genomic Medicine Research Unit, Division of Human Genetics, Department of Pathology, Institute of Infectious Disease and Molecular Medicine, Faculty of Health Sciences, University of Cape Town, Cape Town, South Africa

**Keywords:** Genetics, Gene therapy, Diseases, Visual system

In 2017, the fields of ophthalmology and ocular genetics achieved a major milestone, when Luxturna® (Spark Therapeutics Inc.) became the first ocular gene therapy on the market, approved by the US Food and Drug Administration and later the European Medicines Agency. During the clinical trials stage, a new tool, multi-luminance mobility testing (MLMT), was developed to measure therapeutic effect, and showed striking improvements in patients’ ability to navigate an obstacle course under different light conditions after treatment [1]. A single administration of Luxturna®, via subretinal injection in each eye, improves functional vision in patients with severe vision loss, and a quick internet search unearths poignant success stories of patients describing how their daily lives have improved after receiving the gene therapy.

The excitement surrounding this advancement is tempered by the facts that (1) Luxturna® gene augmentation therapy benefits only those patients with vision loss specifically due to biallelic mutations of the *RPE65* gene; (2) it took 10 years from the initiation of Phase I clinical trials in 2007 [2–5], before it reached the market in 2017; (3) the treatment currently costs $850,000 ($425,000 per eye); and (4) subsequent to authorisation for market use, chorioretinal atrophy was identified as an adverse event through the long-term monitoring of patients who have received the gene therapy [6, 7]. Whilst visual function does not appear to be significantly impacted by this complication, the aetiology remains unknown and thus presents a concern.

Notwithstanding these considerations, eye diseases remain at the forefront of personalised treatment innovation. In 2020, the BRILLIANCE trial (ClinicalTrials.gov ID NCT03872479) was the first to insert CRISPR-Cas9 directly into the human body [8]. This pioneering work was again carried out to address congenital blindness; the gene-editing therapeutic EDIT-101 was designed to target a specific intronic variant of the *CEP290* gene, and the Phase 1/2 trial demonstrated ‘proof of concept and favourable safety profile’ [9].

It is estimated that one third of blindness amongst the working-age population has a genetic basis. These heritable conditions primarily affect the photoreceptor cells (the rod and cone cells) of the retina and/or the retinal pigmented epithelium. The retina functions to translate light signals into electrochemical signals which the brain then interprets, so these inherited retinal disorders (IRDs) result in familial forms of vision loss which can progress to total blindness. As a group, IRDs are one of the most genetically and clinically heterogenous medical conditions, with ~280 associated genes. Finding the causal genetic variant(s) for a patient or family is like searching for the proverbial needle in a haystack.

The number of genes involved in IRDs is perhaps unsurprising when one contemplates the speed at which the retina needs to respond to minuscule changes in light, movement, shadows, objects, etc. for normal vision to occur. The visual transduction pathway, which is constantly converting photons of light into electrochemical signals, is supported by the visual cycle which is rapidly replenishing essential compounds. The retina is therefore an extremely sophisticated, specialised, and metabolically active layer of tissue; mitochondrial genes are highly expressed, splicing activity is elevated when compared to other tissues, and there is unparalleled gene transcript diversity due to novel splicing events/alternative splicing [10]. Taken together, this explains why the retina is particularly sensitive to dysfunction or disequilibrium. Furthermore, unlike other Mendelian disorders, IRDs have a low impact on reproductive fitness, allowing mutations to be passed on to subsequent generations (often prior to disease onset in autosomal dominant forms). It is believed that this is the main reason for the vast number of IRD genes and mutations. The hundreds of genes also reduce the likelihood of two partners/parents in the general population carrying pathogenic variants in the identical gene, despite an extremely high global carrier frequency (estimated ~1 in 3 individuals for autosomal recessive IRDs [11]).

Irrespective of the genetic complexity of IRDs, there are several reasons why the eye is an ideal organ for gene therapy or gene-based interventions. First, the eye is small, needing only a small volume of treatment to be administered. Second, it is compartmentalised (with the blood-retinal barrier maintaining the microenvironment), and immune privileged, therefore there are little-to-no unwanted off-target effects and limited immunological response. Third, the target tissue is easy to access, and the ocular media is transparent. Fourth, eyes are paired, which was leveraged during the early *RPE65* gene augmentation trials; only the worse affected eye was treated and the ‘better’ eye untreated (as an internal control and to preserve remaining vision of participants should complications arise from the treatment). Interestingly, 1.5 years after administration, the treated eyes had better visual acuity than the uninjected eyes [12]. Finally, there are measurable outcomes/endpoints that can be used to evaluate efficacy, such as the MLMT and other non-invasive diagnostic technologies, to detect tangible improvement of vision. It should be noted that retinal neurons (including the photoreceptors) are post-mitotic, which present a double-edged sword: on the one hand, the treatment agent is not diluted through further cell division, on the other hand, there is a narrow window of opportunity for treatment whilst enough cells remain viable.

Because the eye is so amenable to therapeutic interventions, there are an increasing number of gene therapy trials for IRDs. There have been at least 43 trials using AAV-based delivery (the preferred vector for retinal gene therapy) alone [13], however many different modalities are being applied in this rapidly evolving field [14]. Gene augmentation/replacement is the key approach for (a) rescuing the haploinsufficiency in autosomal recessive and X-linked recessive IRDs, (b) enhancing the supply of survival/neuroprotective factors, and (c) converting non-light-sensitive retinal cells into artificial photoreceptors through the expression of light-activated proteins, i.e., optogenetic therapy. On the other hand, for the ‘gain of function’ or ‘dominant-negative’ variants underlying autosomal dominant IRDs, gene ablation and replacement, gene-editing and base-editing are being investigated. In addition, antisense oligonucleotide (AON) and small interfering RNA technologies are being applied to modulate splicing and gene expression [14]. Furthermore, CRISPR/Cas9-mediated cellular reprogramming has been shown to convert a mutation-sensitive cell type to a mutation-resistant cell type, i.e., a rod photoreceptor to a cone-like photoreceptor [15, 16], and induced pluripotent stem cell technology is being developed to regenerate retinal cells [14]. With so many tactics being explored, and the vast phenotypic and genetic heterogeneity displayed by IRDs, it is likely that combined approaches will ultimately be used.

For gene-based therapies, a genetic diagnosis is an obvious prerequisite for treatment. In fact, having the ‘correct’ mutation may be necessary for eligibility, e.g. sepofarsen, an RNA-based AON specifically targets c.2991+1655A>G in the *CEP290* gene [17]. For other interventions, a specific type of mutation may be acceptable, e.g. translational read-through inducing drugs have been tested for nonsense variants [14]. Even for ‘gene-independent’ or ‘non-genetic’ treatments, the underlying genetic diagnosis could be an important factor in establishing efficacy, yet molecular testing is not universally available. This naturally highlights the imperative of improving access to genetic testing in Africa to ensure access to precision ophthalmology.

As the ancestral population of all humans worldwide, Africans display vast genomic diversity [18], with more variation than non-African individuals [19–22], thereby allowing identification of novel mutations and genes, and reducing the misclassification of genetic variants [23]. The diagnostic challenge of identifying pathogenic IRD variants in African genomes would be more akin to looking for a gold needle in a haystack of silver needles. Nevertheless, there is a moral obligation to perform IRD genetic screening on the African continent, so that these patients are not excluded from the gene-based therapeutics that are rapidly emerging internationally. As the Rare Disease Working Group of the H3Africa Consortium notes: ‘data disparity ultimately results in health disparity’ [24]. The identification of panethnic variants, multiethnic variants, population-specific variants and founder effects is important for several reasons [14]. First, recurrent variants could be targeted for efficient screening in low resource environments where comprehensive genetic testing may be financially prohibitive. For example, homozygous *BBS10* [25] and *MYO7A* [26] founder mutations are major contributors to Bardet-Biedl syndrome and Usher syndrome in South Africa, respectively, and both have ocular symptoms. Second, the existence of such ‘common targets’ should impact the design of therapeutic interventions that preserve and restore vision. Furthermore, given the history of the so-called Bantu expansion and global slave trade, ancient founder lineages may also be extrapolated to the African diaspora and other global populations.

Perpetuation of global healthcare inequity though imbalanced research is a real concern in the age of individualised/precision medicine. Recent calculations using publicly available sequence data of 187 autosomal recessive IRD genes from six major world populations, predicted that over 60% of all patients with biallelic *RPE65* gene mutations were from the ‘African’ population, whilst only 9% were Europeans [11]. It should be noted that the ‘African’ cohort essentially comprised African–American individuals, thus the prevalence of *RPE65* mutations may not be applicable across the diverse genomic landscape of Africa. Nevertheless the Luxturna® treatments would likely be a financial impossibility for most patients who need it. Not to mention that African patients have yet to be identified to be eligible for this therapy, due to the costs of genetic testing and the lack of ophthalmic genetics research on the continent.

Precision ophthalmology requires research, collaboration, awareness, education and advocacy. There is a need for (a) genetic literacy amongst current ophthalmology practitioners in Africa, (b) capacity building of African genomic scientists, as well as (c) support from ocular genetics specialists (both local and global) and trained genetic counsellors. Engaged communities of patient-led support groups can influence governments and motivate decision-makers in the global pharmaceutical industry to commit to ensuring that treatments are not reserved for those with financial means. Working together, these stakeholders can promote equitable access to gene-based therapies, and Africa can advance from ‘recipient’ to ‘contributor’ of therapeutic developments.
